# When Heavy Metals Weigh on the Mind: A Case Report of Neuropsychiatric Manifestations of Lead Toxicity From a Retained Bullet

**DOI:** 10.7759/cureus.83574

**Published:** 2025-05-06

**Authors:** Christian Galindo, Juliana Ortiz, María Fernanda Angel, Stephanie Ospina Lopera, Julio Franco

**Affiliations:** 1 Psychiatry, Corporación Universitaria Remington, Medellín, COL; 2 Medical School, Corporación Universitaria Remington, Medellín, COL; 3 General Medicine, Corporación Universitaria Remington, Medellín, COL

**Keywords:** clinical case report, heavy metal toxicity, lead poisoning, neuropsychiatric symptoms, retained bullet

## Abstract

Lead toxicity is a rare but potentially serious clinical condition that can manifest with a wide range of neuropsychiatric and systemic symptoms. We present the case of a 29-year-old male patient from Medellín, Colombia, who experienced delayed-onset neuropsychiatric symptoms secondary to chronic lead exposure from a retained bullet fragment in the left ankle, following a firearm injury sustained 13 years earlier. The patient initially presented with essential tremor in both upper and lower limbs, nausea, vomiting, subjective fever, a metallic taste in the mouth, mood disturbances, significant unintentional weight loss, and chronic mixed-type ankle pain with localized edema. A blue line on the gums (Burton’s line) was observed on physical examination, raising suspicion for lead poisoning. Blood lead level testing confirmed severely elevated levels, supporting the diagnosis of chronic lead intoxication. Additional laboratory tests revealed moderate to severe normocytic anemia and transiently elevated serum creatinine. Despite the persistence of symptoms for approximately three years, diagnosis and treatment were delayed. The bullet was surgically removed after approximately seven months of clinical deterioration. The case highlights the systemic impact of chronic lead exposure and the importance of recognizing non-occupational sources, such as retained ammunition fragments, as potential causes. This report emphasizes the need for early recognition of atypical neuropsychiatric presentations, particularly in patients with a history of trauma or exposure to heavy metals. It also reinforces the relevance of integrating clinical findings with occupational and environmental history to guide diagnosis. Lead intoxication remains a significant public health issue, especially in regions with limited regulation or awareness, and should be considered in the differential diagnosis of unexplained systemic and cognitive symptoms.

## Introduction

Lead poisoning (saturnism) remains a persistent global public health issue due to its systemic nature, its toxicity at low concentrations [1], and its continued presence in occupational, military, and residential settings [2]. Although occupational exposure, such as in the battery [3], paint, or smelting industries, has been extensively documented historically [4], less recognized sources can also result in chronic and significant exposure. One such clinically relevant source is the retention of firearm projectiles in the body, particularly when lodged in tissues with high metabolic activity.

Lead, the second most toxic metal after mercury, has no physiological function in the human body. Its high affinity for proteins and its ability to mimic calcium and zinc allow it to interfere with multiple biological processes, including neurotransmission, hematopoiesis, bone [5], and kidney metabolism. Once absorbed via digestive, respiratory, dermal routes, or through retained foreign bodies, lead is preferentially distributed to bones, soft tissues, and the nervous system [6], where it can exert toxic effects even at levels previously considered "safe."

The literature has described that fragments of lodged projectiles in structures such as joints, soft tissues, or metabolically active bones can slowly release lead into the local environment, promoting its systemic absorption [7,8]. This situation becomes more relevant in contexts where surgical removal is not performed due to the absence of immediate symptoms [9]. However, over the long term, insidious clinical manifestations may emerge, complicating diagnosis, especially when neuropsychiatric symptoms predominate [10].

We present the clinical case of a man residing in Medellín, Colombia, who was 29 years old at the time of presentation. He exhibited progressive neurological and psychiatric symptoms, including auditory hallucinations, behavioral disturbances, irritability, and executive dysfunction [11,12], in the context of lead poisoning secondary to the chronic retention of a projectile lodged in his left ankle for the past 13 years. The diagnosis was confirmed by a markedly elevated blood lead level (199.3 µg/dL), accompanied by systemic findings such as essential tremor, nausea, subjective fever, rapid weight loss, and the presence of the classic Burton’s line [13].

This case report aims to highlight the importance of considering non-occupational lead exposure [14], particularly from retained firearm projectiles, in the differential diagnosis of atypical neurological and psychiatric syndromes [15]. Likewise, it underscores the need to integrate clinical findings with appropriate toxicological evaluation and a contextualized medicolegal perspective to avoid underdiagnosing a potentially reversible yet underestimated condition.

## Case presentation

A 29-year-old male patient from Medellín, Colombia, presented with a constellation of progressive neurological and systemic symptoms in 2022. He was single, unemployed, and enrolled in technical studies. He reported no chronic medical conditions. His personal history included tobacco use since the age of 15 years and past consumption of cocaine approximately three times per week for a period of six years, which he voluntarily discontinued more than six years prior to presentation. He denied any use of alcohol or cannabis.

At age 16, in 2009, the patient sustained a gunshot wound to the left ankle during an isolated episode of community violence. The injury resulted in an open fracture of the left hallux, and the bullet remained embedded in the soft tissues. At that time, the patient did not seek medical attention, as the wound did not cause significant discomfort or complications. As a result, no formal surgical intervention or imaging was performed, and the bullet was never extracted. The patient had no relevant past medical, psychiatric, or family history and had never previously consulted any health professional before this case. He reported no prior trauma-related hospitalizations, no known familial psychopathology, and no history of recurrent involvement in violent episodes. From the time of the injury until about two years prior to presentation, he remained asymptomatic and in apparent good health.

In early 2019, he began to experience a slowly progressive neuropsychiatric syndrome characterized by irritability, verbal and physical aggression (self-directed and toward others), tearfulness, emotional lability, anhedonia, and episodic psychotic symptoms including complex auditory hallucinations and paranoid delusions. These episodes were fluctuating, lasting approximately two to three days, after which he returned to baseline functioning.

Following one of these acute psychotic episodes, the family sought psychiatric evaluation, and he was diagnosed with an acute psychotic disorder. He was started on risperidone 1 mg/day, which was maintained during outpatient follow-up and remained part of his treatment at the time of hospital admission. At that time, basic laboratory tests and cranial imaging were within normal limits.

Over the following two years, no further psychotic episodes were documented, but the patient displayed persistent behavioral alterations such as impaired judgment, executive dysfunction, and affective dysregulation, including difficulty concentrating, abrupt mood swings, marked irritability, apathy, and anhedonia. These symptoms progressively interfered with his interpersonal relationships and academic functioning, as noted by his relatives. In December 2021, he experienced general clinical decline with new-onset systemic and neurological manifestations, including persistent fine tremor in the extremities, nausea, occasional vomiting, subjective low-grade fever, chronic metallic taste, mood disturbances, and an unintentional weight loss of approximately 20 kg over one month. He also reported chronic pain of mixed characteristics and edema in the left ankle. His family noted additional changes in behavior such as increased irritability, poor judgment, disorganized thinking, and occasional auditory hallucinations, prompting further medical evaluation.

**Figure 1:**
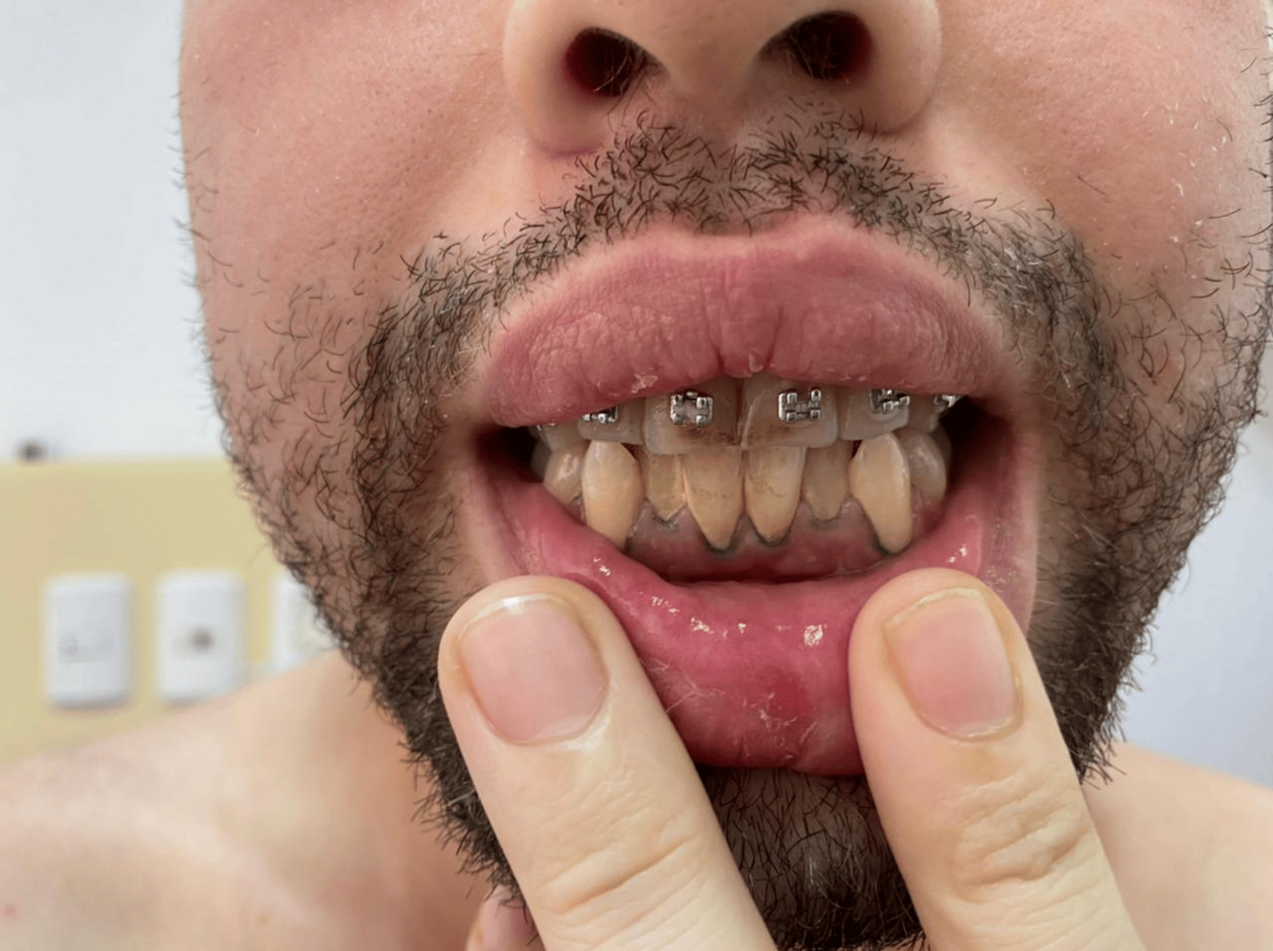
Burton’s line on the gingiva. Bluish-violet pigmentation along the gingival margin, characteristic of chronic lead poisoning, resulting from lead sulfide deposition in the gums.

**Figure 2:**
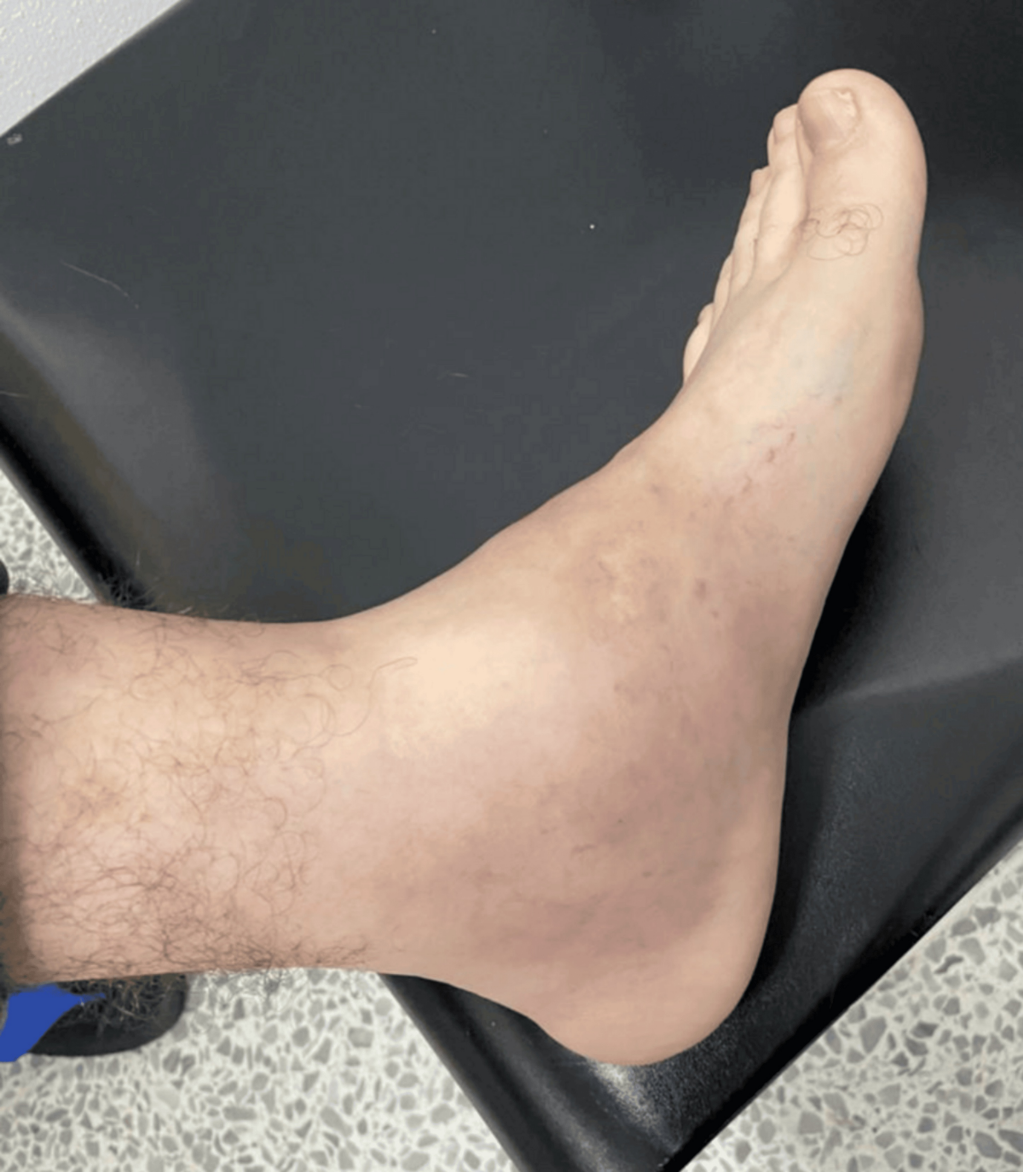
Edematous left ankle with erythema Localized soft tissue swelling and erythema of the left ankle, associated with a retained bullet fragment from a firearm injury over a decade ago, suggestive of chronic inflammatory response

On the present hospital admission, the patient was alert, oriented, and cooperative. Vital signs were stable, with a blood pressure of 109/48 mmHg, heart rate of 68 beats per minute, oxygen saturation of 94% on room air, and temperature of 36.4°C. At admission, the patient was started on midazolam administered as a continuous infusion, hydromorphone 1 mg/0.5 mL every 12 hours, D-penicillamine 250 mg every eight hours, and Ringer’s lactate 0.9% at a rate of 70 cc/hour. The mental status examination revealed preserved orientation but difficulties in semantic memory recall and sustained attention, along with the previously noted fine resting tremor. Physical examination showed generalized pallor and a violet-blue Burton’s line along the gingival margins (Figure 1). Cardiopulmonary and abdominal exams were unremarkable. Examination of the left ankle showed mild soft tissue edema and localized erythema, with no evidence of fistula or purulent discharge (Figure 2). Radiographic imaging showed retained bullet fragments lodged between the calcaneus and talus of the left ankle. 

Laboratory investigations revealed a significantly elevated blood lead level, confirming the diagnosis of severe chronic lead poisoning (saturnism). Hematologic testing showed decreased hemoglobin and hematocrit levels, consistent with moderate to severe normocytic anemia. Serum creatinine was mildly elevated on admission but normalized over the course of hospitalization. Liver enzymes remained within normal limits. A summary of the serial laboratory data, including values at different time points, is presented in Table 1.

**Table 1:** Serial laboratory parameters during hospitalization with reference ranges. MCV: mean corpuscular volume; ALT: alanine aminotransferase; AST: aspartate aminotransferase; ALP: alkaline phosphatase. This table summarizes the patient’s hematological and biochemical profile on days 1, 3, 5, and 7 of hospitalization. Notable abnormalities included persistent anemia, elevated creatinine on admission, and a markedly high blood lead level, which showed progressive reduction. Reference ranges are provided to assist with clinical interpretation.

Parameter	Reference Range	Day 1	Day 3	Day 5	Day 7
Hemoglobin (g/dL)	13.0 – 17.0	7.8	8.2	8.5	9.1
Hematocrit (%)	38.0 – 50.0	27.1	28.5	29.8	31.2
Mean Corpuscular Volume (MCV) (fL)	80 – 100	82	83	85	84
Creatinine (mg/dL)	0.6 – 1.2	1.19	1.02	0.95	0.90
ALT (U/L)	10 – 40	20	18	16	14
AST (U/L)	10 – 40	22	16	17	15
Alkaline Phosphatase (U/L)	40 – 130	78	69	73	76
Blood Lead Level (µg/dL)	<5.0	199.3	170	122	72

In view of the clinical picture and suspicion of toxic metal exposure from a retained foreign body, a plain radiograph of the left foot was performed, which revealed a chronic fracture of the hallux and the presence of a retained projectile in the soft tissue. A multidisciplinary team comprising toxicologists, orthopedic surgeons, and general surgeons was assembled. Following this assessment, CaNa₂ (thylenediaminetetraacetic acid (EDTA)) chelation therapy was initiated at a dose of 1 g/m²/day in 0.9% saline solution for three days.

On August 18, 2022, the patient underwent surgical debridement and lavage of the tarsal/metatarsal region under general anesthesia with fluoroscopic guidance. The procedure was uneventful. Following surgery, the patient remained clinically stable, tolerated oral intake without difficulty, and exhibited no signs of acute toxicity. Significant clinical improvement was noted, including attenuation of neuropsychiatric symptoms and progressive normalization of laboratory values. On postoperative day 7, the blood lead level had decreased markedly from 199.3 µg/dL to 79 µg/dL.

His analgesic regimen was optimized with morphine 2 mg every eight hours diluted in 0.9% saline solution. The patient was discharged on postoperative day 10 in stable condition, showing improvement in mood and behavior. At the time of discharge, his blood lead level had further declined to 9 µg/dL. He was released with scheduled appointments in internal medicine and toxicology for continued clinical follow-up and serial lead level reassessment. He received clear instructions regarding outpatient follow-up and clinical monitoring. At discharge, he continued pharmacological management with acetaminophen 500 mg every eight hours and doxycycline 100 mg every eight hours for five days.

## Discussion

Chronic lead poisoning, also known as saturnism, due to retained bullet fragments, constitutes an uncommon but clinically significant etiology of systemic lead toxicity [8]. Saturnism refers to poisoning caused by lead accumulation in the body [9], typically presenting with multisystemic symptoms including neurological, hematologic, renal, and gastrointestinal disturbances [10]. This mode of exposure may go undetected for years, particularly when the metallic foreign body remains asymptomatic or is not removed.

In the present case, the source of exposure was a gunshot wound sustained over a decade prior, which underscores the latency and insidious nature of lead bioaccumulation. The clinical presentation, ranging from psychiatric symptoms to hematological and neurological disturbances, mirrors findings in prior literature, where retained intrabody projectiles have been identified as persistent endogenous sources of lead release [13]. Once absorbed, lead is distributed to various organs, predominantly binding to erythrocytes and accumulating in bones, where it may reside for decades [14]. This case reinforces the concept of cortical bone serving as a long-term reservoir that continuously reintroduces lead into the circulation, especially under conditions of physiological demineralization or inflammation [15]. 

The patient's progressively worsening symptoms, including behavioral disinhibition, anhedonia, executive dysfunction, and auditory hallucinations, are consistent with previously described neuropsychiatric manifestations associated with chronic lead exposure, particularly in adults with a cumulative burden [16]. The diagnostic challenge lies in the polymorphic presentation of lead intoxication, which often mimics primary psychiatric disorders such as mood disorders, schizophrenia, or post-traumatic stress disorder (PTSD) [17]. The initial misdiagnosis of this patient with an acute psychotic disorder and the absence of toxicological testing highlights the need for a broader differential diagnosis in atypical or treatment-resistant psychiatric presentations. 

Studies have demonstrated that lead's disruption of calcium- and zinc-mediated intracellular signaling and its affinity for neural tissue can result in functional alterations of the prefrontal cortex and basal ganglia [18], regions responsible for mood regulation, impulse control, and executive function. These pathophysiological mechanisms provide a plausible explanation for the observed symptomatology. Additional somatic signs, such as normocytic anemia, persistent metallic taste, and the presence of a Burton's line-served as clinical semiological clues pointing towards heavy metal intoxication [19]. Laboratory findings confirmed the suspicion with a markedly elevated blood lead level (199.3 µg/dL), far exceeding the chelation threshold of 45 µg/dL. 

Although bone lead quantification via K-X-ray fluorescence (KXRF) remains the gold standard for assessing cumulative exposure, its absence in this case does not undermine the diagnostic confidence, given the congruent clinical, laboratory, and historical data. The therapeutic approach included surgical debridement and partial removal of the projectile, which effectively curtailed further exposure. Although chelation therapy was not initiated, it remains a viable consideration depending on the evolution of post-discharge lead levels. The case outcome was favorable, with stabilization of hematologic and renal parameters and gradual improvement of neuropsychiatric symptoms, suggesting that early source control can meaningfully alter prognosis and mitigate long-term sequelae. 

From a broader clinical perspective, this case underscores the importance of incorporating environmental and toxicological etiologies into the differential diagnosis of psychiatric disorders, particularly in patients with a history of violence or trauma. It also advocates for increased clinician awareness regarding non-occupational forms of saturnism. Routine psychiatric evaluation should consider exposure history, particularly when symptoms are atypical, fluctuating, or are refractory to standard treatment. Moreover, the limitations of relying solely on blood lead levels in chronic cases should prompt a more comprehensive diagnostic strategy, including the use of surrogate markers such as zinc protoporphyrin (ZPP) or KXRF when available [19]. Lead interferes with heme synthesis by inhibiting ferrochelatase, resulting in elevated levels of ZPP, which can be measured in blood as an indirect marker of lead exposure. Meanwhile, KXRF provides a non-invasive method to quantify lead content in cortical bone, offering a reliable estimate of cumulative exposure over time [20]. Finally, the integration of this case with previous literature reinforces the significance of interdisciplinary management and the value of nuanced clinical judgment in uncovering reversible toxic-metabolic causes underlying neuropsychiatric symptoms. Such approaches are vital to improving diagnostic accuracy and preventing chronic morbidity in patients affected by insidious environmental toxins.

## Conclusions

This case underscores the diagnostic complexity of chronic lead poisoning when clinical features resemble primary psychiatric disorders. Subtle physical signs and neuropsychiatric symptoms may delay recognition, especially in non-occupational exposures such as retained bullet fragments. A comprehensive, exposure-focused history is essential.

Accurate differentiation between psychiatric, substance-related, and toxic-metabolic conditions is critical. Multidisciplinary intervention, including surgical removal of the source, was central to the patient’s recovery. This report highlights the importance of clinical vigilance and integrative reasoning in identifying reversible causes of cognitive and behavioral decline.
